# Perceived Psychotherapist's Empathy and Therapy Motivation as Determinants of Long-Term Therapy Success—Results of a Cohort Study of Short Term Psychodynamic Inpatient Psychotherapy

**DOI:** 10.3389/fpsyt.2018.00660

**Published:** 2018-12-04

**Authors:** Frank Vitinius, Stephanie Tieden, Martin Hellmich, Holger Pfaff, Christian Albus, Oliver Ommen

**Affiliations:** ^1^Department of Psychosomatics and Psychotherapy, University Hospital of Cologne, Cologne, Germany; ^2^Institute of Medical Statistics and Computational Biology, University of Cologne, Cologne, Germany; ^3^Institute of Medical Sociology, Health Services Research, and Rehabilitation Science, University of Cologne, Cologne, Germany

**Keywords:** depression, follow-up, inpatient psychotherapy, outcome predictors, treatment outcome

## Abstract

**Objective:** Outcome predictors and determinants for treatment outcome of inpatient psychotherapy will be assessed in a follow-up-study. Sociodemographic factors and the level of depressiveness at admission, the perceived psychotherapist's empathy rated by patients and the therapy motivation as possible moderators of treatment outcome (reduction of depressive symptoms) are analyzed.

**Methods:** In a cohort study, the outcome of inpatient multimodal psychotherapy was examined with Beck-Depression-Inventory (BDI) at admission (T1), discharge (T2) and at follow-up (1–3 years after treatment) (T3). Inclusion criteria were: Inpatient psychotherapy between 2007 and 2010 with a duration of at least 1 week and complete data set. The influence on therapy success of (1) sociodemographic factors, (2) the perceived psychotherapist's empathy rated by patients using the Consultation and Relational Empathy Measure (CARE), and (3) the therapy motivation of the patients rated by therapists are examined by means of correlation analysis, distribution comparisons and subsequently logistic regression.

**Results:** Ninety-two (64 females, average age 39 yrs.) of 182 eligible patients participated in the follow-up survey. Duration of inpatient psychotherapy lasted 8.7 weeks ± 3.6 [min. 1, max. 33 weeks]. The perceived psychotherapist's empathy, therapy motivation, education level and depression at baseline had a significant impact on therapy success. Gender, age, and partnership were not significant. The length between discharge and follow-up had no influence on the results. Based on these variables a multiple logistic regression explained 42% of the variation (goodness-of-fit).

**Conclusion:** Due to the shown relevance of the psychotherapist's empathy perceived by patients and the therapy motivation of patients for therapy success, both factors should be considered already at the beginning of the therapy. Consequently, they should be recognized in the context of postgraduate training and education.

## Introduction

The effectiveness of inpatient psychotherapy which in most cases is short-term psychotherapy has already been demonstrated in various studies in psychosomatic-psychotherapeutic acute and rehabilitation clinics with different outcomes (1–6).

Furthermore, numerous follow-up studies (catamnesis studies) showed positive long-term effects of inpatient psychotherapy (5, 7–10).

In recent years, predictors have also been identified in various studies for the treatment success of psychotherapy. Sociodemographic as well as disease and therapy-related factors could be identified as possible moderators of the therapy success. However, some controversial results were found or recognized.

Lambert and Barley (11) summarized the research on therapeutic relationship and psychotherapy outcome. In their research factors as empathy and the therapeutic relationship correlated more highly with outcome than specialized treatment interventions.

Previous studies in inpatient psychotherapy with a mixed patient sample showed no relationship between gender and therapy success (2, 5, 6, 12, 13). The relationship between age of the patient and therapy success is just as inconsistent as the relationship between partnership and therapy success (2, 5–7). Thus, Beutel et al. (5) could show in a comparative study design between inpatient short- and long-term therapy, that the lack of a stable partner is a negative predictor of therapy success in a long-term setting whereas the presence of a stable partnership has no significant effect on the success of inpatient psychotherapy (6, 12).

Riedel (14) showed that patients with a lower education level had a significantly lower therapy success. Hiller et al. (15) reported, that patients with lower education level showed more improvements in their symptoms during inpatient therapy for somatoform disorders than patients with a higher education level. In other studies the level of education had no influence on the therapy success (6, 16).

More obvious is the relationship between patients' therapy motivation and its therapy success. Previous studies have consistently concluded that therapy motivation is a major predictor of therapy success (6, 14, 17–20).

A number of studies have shown that patients who are depressed are significantly more likely to benefit from inpatient psychotherapy (2, 10, 21, 22). Patients with depressive disorder showed better therapy success than patients with other non-somatic diseases (6).

Bassler (7) showed that a lower level of depression at the beginning of therapy proved to be prognostically beneficial for therapy success. In contrast to this, a strong somatic comorbidity has an unfavorable effect on the therapy success (12). To sum it up the present findings on depression are inconsistent.

Patients with positive treatment outcome assessed the relationship with their therapists as good, while patients with worsened outcome also judged the therapeutic relationship more critically (23). The therapeutic relationship in an individual therapy has therefore been described by Sammet et al. (23) as a “relevant treatment component.” Bassler (24) also showed that the relationship between patient and therapist is a relevant criterion for the success of inpatient psychotherapy. In a study of Konzag et al. (25), the patient's evaluation of the therapeutic relationship was also an important prognostic factor for the outcome of inpatient psychotherapy.

The relationship between patient and therapist plays a decisive role for the success of the treatment, regardless of the therapeutic orientation or the school of therapy (26, 27).

Especially, Norcross and Wampold (28) focused on evidence-based therapy relationships. In a research based on meta-analyses, Norcross and Wampold reported several effective relationship elements, e.g., empathy and alliance in psychotherapy.

A positive therapeutic relationship is characterized by “empathic understanding” (29).

Berger (30) showed that the empathy of the therapist in the sense of “empathic compassion” as well as respect is of great importance for a successful psychotherapy. Similarly, Malin and Pos (31) and Watson et al. (32) showed the impact of empathy for the psychotherapeutic success.

Some studies on empathy perceived by the patient that is reflected in psychophysiological synchronization, e.g., Messina et al. (33), showed that shared psychophysiology can indeed represent an unbiased measure of empathy in terms of synchronization between patient and therapist. In particular, the study of Messina et al. (33) took into account the training of psychologists, showing that the more trained is the psychologist, the higher is the synchronization in clinical dyad.

Palmieri et al. (34) showed that psychophysiological synchronization [already proven to be a correlation of the patient's perceived empathy by the therapist as demonstrated by Messina et al.(33)] increases in the clinical dyad when the psychotherapist receives an induction to a mental state related to secure attachment. This means that, in addition to training, also other strategies related to positive mental states can increase the empathy perceived from patient by the therapist.

In a study of Barnicot (35) clinicians' empathy was significantly associated with lower depression severity during treatment.

However, the present findings on empathy as a predictor of therapy success are contradictory and depend among other factors on the measures used (36–38). The results of two meta-analyses (36, 39) regarding the relationship between empathy and psychotherapeutic outcome showed that empathy perceived by patients could significantly better predict therapy success than empathy assessed by therapist or observer. Empathy as a predictor of therapy success and as a determinant of quality was described not only especially for psychotherapeutic settings (40, 41), but also described for medical care generally (42).

Abbas et al. (43) published a Cochrane Review on short-term psychodynamic psychotherapies for common mental disorders. They identified different outcome measures for therapy success like general, somatic, anxiety and depression reduction.

The present study aims to determine predictors and determinants for therapy success in inpatient psychotherapy measured by reduction of depressiveness. Empathy of the therapist perceived by patients has not—or without using a validated questionnaire—been considered in previous studies of inpatient psychosomatic treatment. Therefore, in addition to sociodemographic factors such as gender, age, education, partnership, depression at beginning of therapy, therapy motivation, therapist's empathy perceived by patients is to be investigated as a possible influencing factor using the internationally validated CARE-questionnaire.

## Materials and Methods

### Design

Consecutive patients of the psychotherapy inpatient ward of the Department for Psychosomatics and Psychotherapy, University Hospital of Cologne, were included in a retrospective cohort study including a follow-up assessment. Standardized patient questionnaires at T1 (inpatient admission), T2 (a few days before discharge) and T3 (follow-up, i.e., 10–38 months after discharge) were used. The psychotherapeutic intervention—according to the operations and procedures key for medical interventions (OPS key number 9-63 for so-called psychosomatic-psychotherapeutic complex treatment in German departments of psychosomatics and psychotherapy)^1^—consists in our clinic of the following elements:

- frequent psychodynamic psychotherapy in an individual-setting (2 sessions à 50 min weekly)
- psychodynamic psychotherapy in a group-setting (2 sessions of 60 min per week)
- psychodynamic movement psychotherapy in a small group-setting (2 sessions à 60 min a week)
- art therapy in a small group-setting (1 session à 125 min per week)
- music therapy in a small group-setting (2 sessions à 50 min per week)
- daily team rounds
- weekly visit of the head of the department with an indication conference
- participation in a somatic consultation session, which takes place at least once a week
- integration into a daily ward routine as well as regular therapeutic nursing care
- sports therapy, physiotherapy
- if necessary, disorder-specific treatments (e.g., pain, PTSD), diaries, imaginative exercises, mindfulness exercises or disturbance-specific elements in other disorders such as eating disorders or anxiety disorders.

From 2007 until 2010 the psychotherapists of our department are four long-term experienced physicians for psychiatry/psychosomatics with additional supervision. The study was approved by the Ethics Commission of Cologne University's Faculty of Medicine (code 09-263, 28th April, 2010). All subjects gave written informed consent in accordance with the Declaration of Helsinki.

### Study Population - Inclusion and Exclusion Criteria

Inclusion criteria were a hospital-stay of minimum 1 week (in the period from February 1, 2007 to December 31, 2010) as well as completed questionnaires for admission, discharge and follow-up for all questions relevant for the study. Subsequent stays of patients who were readmitted or who have had multiple inpatient stays during this period were excluded.

All questionnaires at T3 were sent to the patients by mail. In case of non-reply, a second postal sending of the questionnaires and repeated telephone call attempts were carried out according to the Dillman method (44).

### Instruments

#### Psy-BaDo

At the time of hospital admission the patients completed the Psy-BaDo basic documentation on sociodemographic data according to Heuft and Senf (45). The Psy-BaDo therapist's questionnaire was also completed at admission. With this questionnaire, the therapist rated the patients' therapeutic motivation on a scale from 0 to 4 (0 = not motivated to 4 = very motivated). In addition, the questionnaire contains questions regarding the duration of complaints, referring clinician or institution, pre-treatment aspects, severity of impairment, symptoms according to ICD-10 and functional level.

#### BDI

To assess the severity of depression, the German version by Hautzinger et al. (46) of the Beck Depression Inventory (BDI) (47) was used at all three measurement times. The BDI is a valid, reliable and objective 21-question self-report inventory. It measures cognitive as well as affective, somatic, motivational and behavioral aspects. The symptoms of the last 8 days are rated by the patient on a four-level scale from 0 (nonexistent) to 3 (strong). For evaluation, the sum of the 21 items (0–63 points) is calculated and the degree of severity of the depression assessed. There are no depressive symptoms for sum scores below 10 points, 10–18 points indicate mild, 19–29 points moderate, and 30 or more points a severe depressive symptomatology.

#### Consultation and Relational Empathy Measure (CARE)

The Consultation and Relational Empathy Measure (CARE) was used to measure patients' perceived empathy of the therapist at the time of T3. The one-dimensional CARE is a 10 item measure tool with good psychometric properties (48, 49). Four empathic components are described and assessed: emotive, moral, cognitive and behavioral aspects. Thus, the concept of empathy has to be distinguished from the purely emotional definition as well as from the concept of sympathy. Empathy is seen as a professional and learnable therapeutic ability (48, 50). Neumann et al. (50) developed a German version of CARE.

### Statistics

Nearly all of our patients in our heterogeneous sample suffered from depressiveness. Therefore, we decided to use depressiveness as marker of therapy success. The change in BDI (BDI^ΔT1/T3^) was chosen as the main therapy outcome. Mean values of the BDI sum scores at admission (T1), discharge (T2), and follow-up (T3) were compared with the *t*-tests for paired samples.

The following variables were examined as possible predictors of therapy success: gender, age, level of education, partnership status, severity of depression (BDI at T1), therapy motivation (at T1) and patients' perceived empathy of the therapist at T3 (CARE Measure). To test if the BDI pre-treatment score contributes to a high percentage of explained variance a second regression analysis was performed without BDI at baseline. For identification of differences between patient groups with successful and less successful/unsuccessful therapy and to identify any predictors for this, the dependent variable (BDI^ΔT1/T3^) was dichotomized by means of a typical (51) 1:2-split into two groups: 33.3% (no or only slight improvement in depressive symptoms) vs. 66.7% (clinically relevant improvement in depressive symptoms).

Correlations between any two variables were summarized by Spearman's rho correlation coefficient. We chose spearman's rho due to the apparent non-normality of data distributions. While equidistant scaling may aid in interpretation, it is not required for the coefficient to be a valid measure of the strength of a monotone relationship (between any two variables). Differences in distribution between any two variables were evaluated by Mann-Whitney *U*-test or Pearson's chi-square test, contingent on distributional characteristics. Subsequently, a stepwise logistic regression model was fitted with variables entered in two separate blocks. In the first block, the variables gender, age, level of education, partnership status, severity of depression (BDI at T1) and the duration of follow-up were entered. Therapy motivation (at T1) and patients' perceived empathy of the therapist (CARE Measure at T3) were entered in the second block (final logistic regression model). Additionally, bivariate analyses were performed to examine whether the patients who participated in the follow-up survey (T3) were different from those who had not participated. All analyses were performed with SPSS Statistics for Windows, version 22 (IBM Corp., Armonk, NY, United States). We visually checked the normality of data distributions as a requirement for using parametric tests.

## Results

### Sample

Between 2007 and 2010 199 psychotherapy inpatients were treated.

**Figure 1:**
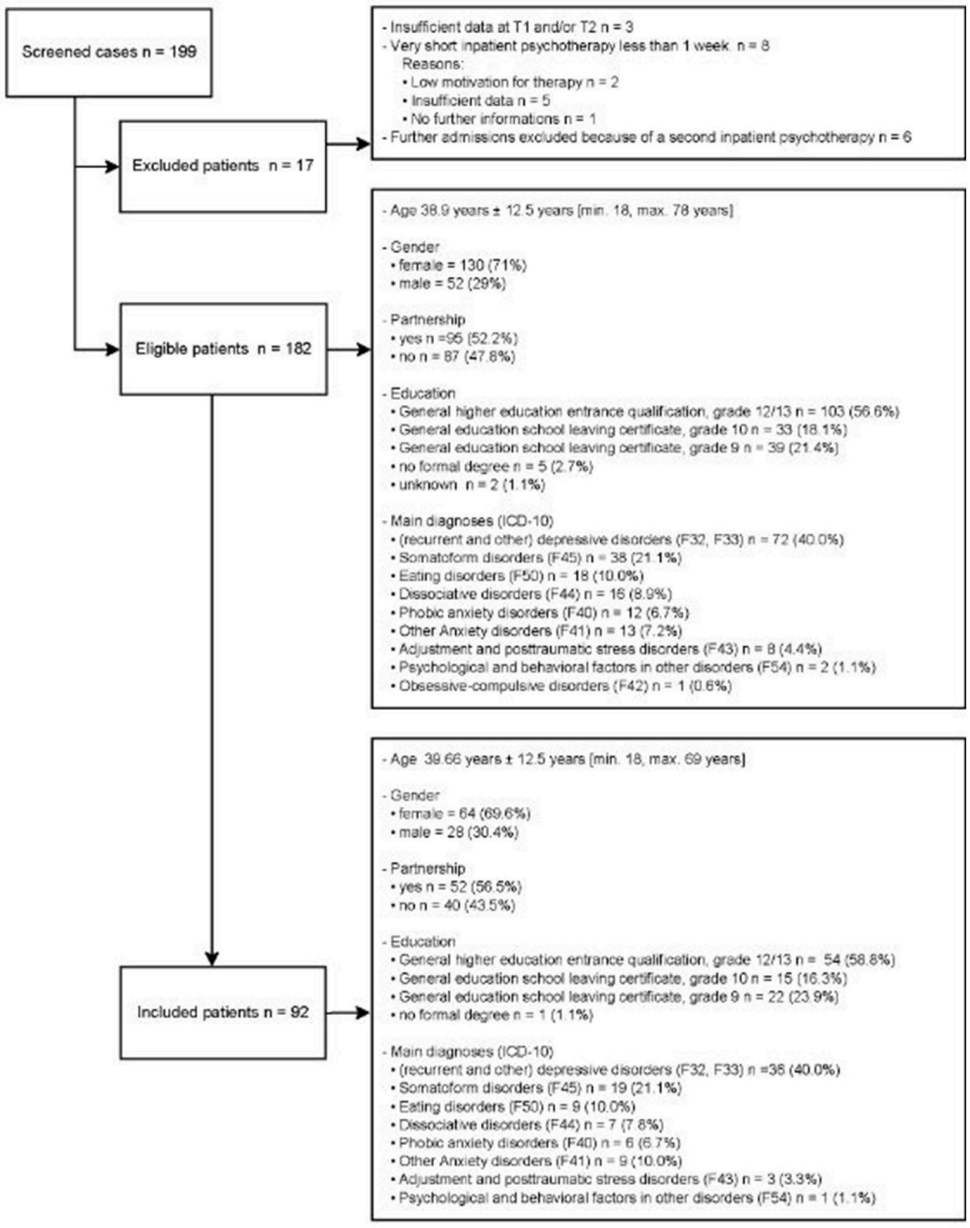
Study sample.

In this cohort study 182 Patients are eligible (Figure 1). Seventeen patients have refused to participate in this study, 3 patients have died because of somatic diseases (no suicide), from 15 patients the new address was unknown and 55 patients did not respond. In the end, we could include 92 patients.

The sociodemographic and clinical characteristics of the sample are depicted in Figure 1.

In this study the patients have suffered from different mental disorders (Figure 1). The duration of inpatient psychotherapy lasted 8.7 weeks ± 3.6 [min. 1, max. 33 weeks]. The follow-up assessment has taken place 19.2 ±7.8 months [min. 10, max. 38 months].

After data cleaning 86 of 92 patients could be included in the analysis, i.e., 47.3% of the eligible patients. There are no significant differences regarding gender, age, education level, partnership status, therapy motivation, severity of depressive symptoms and duration of the follow-up between the patients who have participated in the follow-up and the patients who have not participated.

### Dependent Variable (Improvement of Depressive Symptoms)

Nearly all patients showed depressive symptoms at the beginning of the treatment. Only 13 patients (7.6%) have BDI scores in the normal range. These patients were not excluded from analysis because an impairment of depression during the treatment was possible.

There was an unequivocal improvement of BDI sum scores between admission and discharge (BDI^Δ*T*1/*T*3^; *p* < 0.001). At admission (T1) 92.4% of the patients (*n* = 159) revealed depressive symptoms in the BDI (42 patients [24.4%] mild, 75 patients [43.6%] moderate and 42 patients [24.4%] severe depressive symptoms) with a mean score of 23.1 ± 9.83 [0–50] and a median of 23.0. At discharge 46.7% of the patients (*n* = 72) were no longer depressed, 31.2% (*n* = 48) showed mild, 16.9% (*n* = 26) moderate and 5.2% (*n* = 8) severe depressive symptoms.

**Figure 2:**
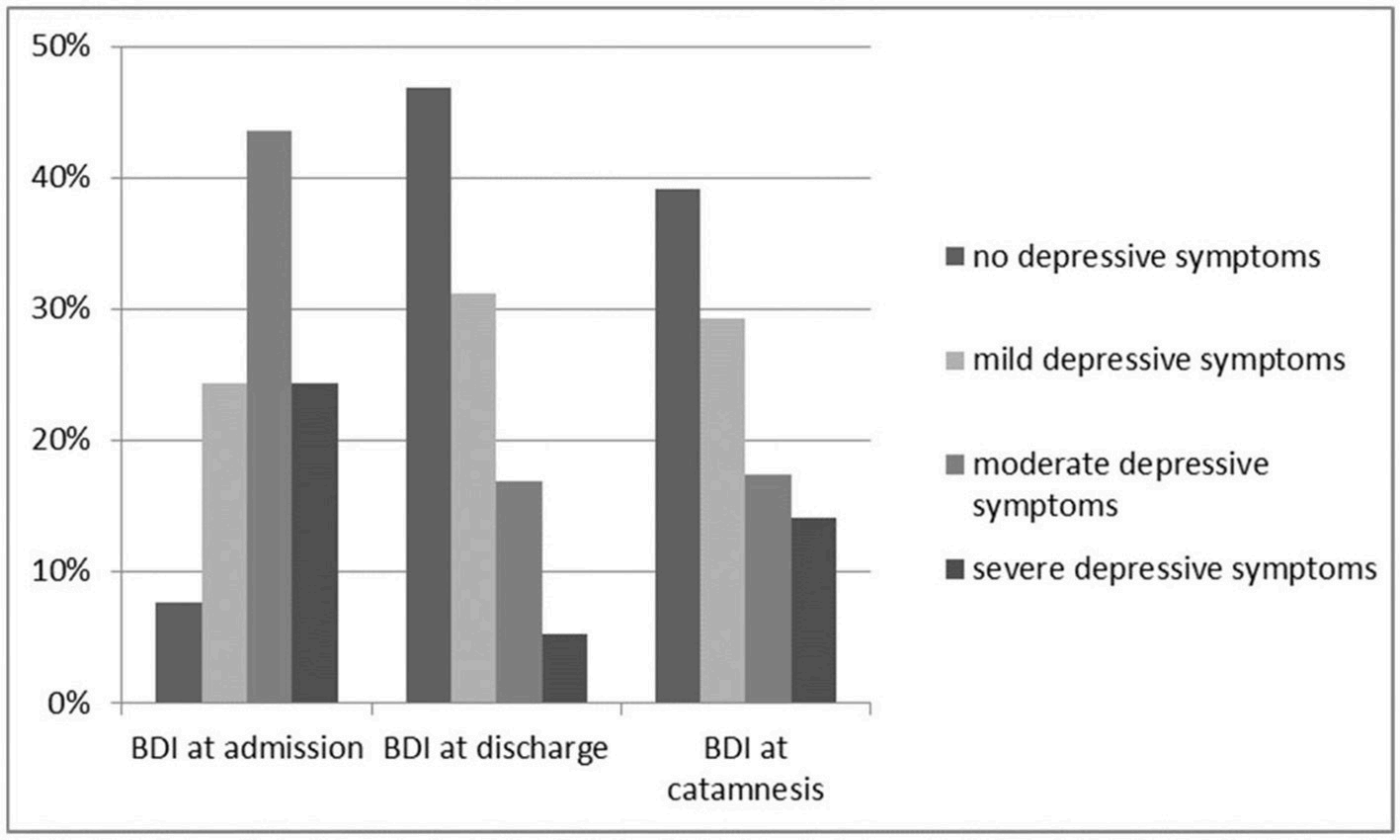
Change of BDI scores.

At T3 some patients demonstrated a small increase of depression. 39.2% of the patients (*n* = 36) were below the threshold regarding depressive symptoms, 29.3% (*n* = 27) revealed mild depressive symptoms, 17.4% (*n* = 16) moderate and 14.1% (*n* = 13) severe depressive symptoms (Figure 2).

Between admission and discharge a strong improvement of BDI sum scores could be demonstrated (*p* < 0.001; mean 10.52 ± 8.76, 95% CI 9.1; 11.9). Between admission and T3 (*p* < 0.001, mean 7.79 ± 11.32, 95% CI 5.4; 10.2) and between discharge and T3 (*p* = 0.005, mean −2.93 ± 9.10, 95% CI −4.9; −0.9) a significant improvement of BDI sum scores could be demonstrated.

### Predictors and Determinants of Therapy Success

#### Correlation Analyses

Besides age, gender, education, existence of partnership and severity of depressive symptoms at the beginning of the therapy the variables therapy motivation and perceived empathy of the therapist at T3 and the BDI sum score between admission (T1) and follow-up (T3) were included in the correlational analyses. There were significant correlations between the outcome variable BDI^ΔT1/T3^and the BDI sum scores at admission (Spearman-Rho: *P* = 0.024, *r* = −0.240), education (Spearman-Rho: *P* = 0.023, *r* = 0.242), therapy motivation (Spearman-Rho: *P* < 0.001, *r* = −0.368) and patient-reported empathy of therapist (or empathy of therapist assessed by patients (Spearman-Rho: *P* = 0.028, *r* = −0.237).

#### Bivariate Analyses

Results of bivariate analyses revealed significant differences concerning education level, level of BDI sum score at admission, perceived empathy of therapist and therapy motivation (Table 1).

**Table 1:** Comparison of outcome variables “no/small improvement“ vs.” improvement of BDI sum score (BDI^ΔT1/T3^).”

		**Course of BDI sum score between** **admission and follow-up**		***P*-value**
		**No/small improvement or impairment** **(*n* = 28)**	**Clear improvement** **(*n* = 60)**	
Gender^a^				0.386
	Male (29.5%)	10 (35.7%)	16 (26.7%)	
	Female (70.5%)	18 (64.3%)	44 (73.3%)	
Partnership^a^				0.133
	Yes (54.5%)	12 (42.9%)	36 (60.0%)	
	No (45.5%)	16 (57.1%)	24 (40.0%)	
Education^a^				0.023^*^
	High school (60.2%)	12 (42.9%)	41 (68.3%)	
	Less than high school (39.8%)	16 (57.1%)	19 (31.7%)	
Age^b^	Mean (SD)	40.8 (12.8)	38.2 (12.1)	0.266
BDI at admission^b^	Median (IQR)	17.8 (12.1,26.8)	24.0 (19.0,29.4)	0.025^*^
Empathy: CARE-sum score^b^	Median (IQR)	4.2 (3.9, 4.6)	4.6 (4.0,5.0)	0.029^*^
Therapy motivation^b^	Median (IQR)	2.5 (2.0, 3.0)	3.0 (3.0,4.0)	0.001^***^

a *χ^2^-Test for dichotomous variables*.

b *Mann-Whitney U-test for continuous variables*.

* p ≤ 0.05

and

*** *p ≤ 0.001*,

*IQR ‘interquartile range', 25th to 75th percentile*.

A moderator analysis was conducted to exclude a potential bias driven by depression severity at T1 on therapy motivation and perceived empathy of therapists. A possible relationship could be that patients with severe depressive symptoms when compared with patients who have a lower level of depression might perceive a lower level of therapist's empathy and/or a lower therapy motivation.

Therefore, a further dichotomous outcome variable was calculated to conduct an extreme group comparison: 25% of the worst results of change of BDI scores (missing improvement or impairment of BDI sum scores) were compared with the remaining 75% in regard to therapy motivation and CARE sum scores in Mann-Whitney-U-Tests. There were not any significant differences between both groups. Therefore, it seems that the severity of depression did not relevantly impact on the assessment of therapeutic empathy and therapy motivation.

#### Logistic Regression

In the first step of logistic regression analysis the sociodemographic parameters which are depicted (Table 2) could explain nearly 25% of the variance of therapy success “improvement of BDI sum score” (χ^2^ = 17.468, *df* = 6, *p* = 0.008, Nagelkerke *R*^2^ = 0.256), respectively, nearly 14% without BDI at baseline in the regression analysis (χ^2^ = 8.944, *df* = 5, *p* = 0.11, Nagelkerke *R*^2^ = 0.138). In the second step of logistic regression analysis the variables therapy motivation and perceived empathy (CARE sum score) were incorporated in the model. This model could explain nearly 42% of the variance of therapy success (χ^2^ = 13.855, *df* = 2, *p* = 0.001, Nagelkerke *R*^2^ = 0.426), respectively nearly 37 % without BDI at baseline in the regression analysis (χ^2^ = 17,898, *df* = 2, *p* < 0.001, Nagelkerke *R*^2^ = 0.374).

**Table 2:** Logistic regression with BDIΔ T1/T3 as dependent variable (*n* = 86).

	**Step 1**			**Step 2**		
	**OR**	**95% CI**	***P*-value**	**OR**	**95% CI**	***P*-value**
Gender [f vs. m]^***^	1.909	0.608–5.989	0.268	1.881	0.512–6.908	0.341
Age [years]	1.017	0.970–1.066	0.487	1.039	0.984–1.098	0.170
Education [less than high school vs. high school^***^]	0.221	0.070–0.703	0.011^*^	0.254	0.070–0.916	0.036
Partnership [no vs. yes]^***^	0.401	0.134–1.194	0.101	0.321	0.093–1.107	0.072
BDI at T1 [0–63 points]	0.917	0.861–0.976	0.007^**^	0.929	0.865–0.997	0.042
Length of follow-up [months]	0.977	0.910–1.049	0.519	0.943	0.864–1.028	0.184
Therapy motivation [0–4 points]				0.35	0.168–0.731	0.005^**^
CARE sum score [0–5 points]				0.314	0.116–0.849	0.023^*^

* *p ≤ 0.05*,

** *p ≤ 0.01*,

*** *Reference category is given second*.

If the therapy motivation of the patient increased by one unit, the relative odds decreased by 61% that a person is part of the high risk group (worst third/ group “1”) (0.385–1 = −0.615).

If the perceived psychotherapist's empathy rated by the patient increased by one unit, the relative odds decreased by 65% that a person is part of the high risk group (worst third /group “1”) (0.346–1 = −0.654).

The entire model could predict 87.9% of the patients correctly who achieved an important improvement of depressive symptoms, and 57.1% of the patients who experienced no or only a small improvement. The quality of the model amounted to 77.9% of correctly classified cases. A predicted probability from the (multiple) logistic regression of 0.5 or greater was considered to indicate therapy success, thus 0.5 was chosen as the threshold for classifying cases.

## Discussion

This study aimed to detect predictors of therapy success. The effective treatment of depressive symptoms represents an important goal of the whole treatment and impact on the results of the therapy (2, 6, 15, 52, 53). Therefore, the change of depression over time has been chosen as dependent variable because nearly all included patients revealed depressive symptoms at admission.

Beside sociodemographic factors the perceived empathy of the therapist and the therapy motivation of the patient were assessed as possible predictors. Both attributes revealed a significant impact on therapy success. The first time we talk about empathy in psychotherapy corresponds to citations (29–42) in introduction, and some are recited here. The results corresponded to two meta-analyses in which empathy was assigned to have an impact of 10%, respectively 9% on therapy success (36, 39). Comparably to other studies concerning inpatient psychotherapy with mixed patient population the sociodemographic variables gender, age, education and partnership were not significant (2, 5–7, 12, 16). The above described final logistic regression model explained 42% of the variation. To control the impact of depression at baseline we excluded this variable and calculated a further regression analyses. The perceived empathy of the therapist and the therapy motivation still remained significant. The explained variation was likewise quite high (37%). Goodness-of-fit of the second step amounted to 80%, respectively nearly 75% correctly classified cases (model with respectively without BDI at baseline) which seems a very satisfiable value.

The innovative contribution of our study consists in the first-time use of “Consultation and Relational Empathy Measure” (CARE) for inpatient psychotherapy. Until now CARE was used exclusively in the somatic context.

Our study demonstrates that perceived empathy reveals a high significant predictor for the therapy success of multimodal inpatient psychotherapy: The higher the perceived empathy the better the therapy success. Earlier studies aiming at the impact of therapist's empathy on therapy success used other instruments like the Barrett-Lennard Relationship Inventory (BLRI) (31, 32, 39). Watson et al. (32) assessed the impact of perceived empathy assessed with the BLRI by depressed outpatients who were treated with cognitive behavior therapy.

Contrary to the studies of Konzag et al. (25, 54) who showed that empathy has no impact on the therapy success our study clearly demonstrates significant effects. However, Konzag et al. ascertained empathy by the therapists themselves and not from the point of view of the patients.

This discrepancy corresponds to the results of a meta-analysis of Elliot et al. (39) regarding the relationship between empathy and outcome in the psychotherapeutic context. They found that empathy which is perceived by patients could significantly better predict therapy success than empathy assessed by therapist or observer. Malin and Pos (31) also showed that therapists‘ expressed empathy significantly affected the outcome of therapy of depression (reduction of depressive symptoms). Watson et al. (32) showed a significant direct relationship between therapists' empathy and improvement of depression.

We could demonstrate that the application the “Consultation and Relational Empathy Measure” (CARE) is a useful tool in the measurement of psychosomatic and psychotherapeutic therapy success. Comparable to the usage of CARE in a somatic setting (50, 55–58) we could show that the empathy of the physician respectively of the therapist represents a strong influencing factor of the therapy success. On the other site it is quite important to perform research on empathy in somatic diseases and in the health system, too. For example, Decety and Fotopoulou (59) examined why empathy has a positive impact on others and briefly reviewed the various effects of empathy on health outcomes in the domain of medicine. Di Blasi et al. (60) showed that physicians with an empathic manner are more effective. According to a meta-analysis of Kelley et al. (61) the patient-clinician relationship has a significant effect on healthcare outcomes.

The therapy motivation of the patients—assessed by therapists at the beginning of the treatment—was a further important predictor of therapy success in our analysis. This result is in accordance with previous studies (6, 14, 17, 19, 20). Bleichhardt et al. (18) detected therapy motivation as positive predictor of therapy success. Klauer et al. (62) and Zwerenz et al. (63) investigated patients who terminated the inpatient psychotherapy ahead of time. At the beginning of the therapy these patients are strongly less motivated than other patients. It should be assessed in further studies whether initially weak motivation could be positively influenced in the first therapy sessions and could be interrelated to the therapy success (14, 19). Therefore, therapists should aim at a “positive sensitizing” of the patient for his/her psychotherapy already at the beginning of the therapy (17) to reduce potential prejudices against therapeutic measures. This should be done with an open, empathetic attitude in order to modify cognitive attitudes and to reduce existing anxieties of psychotherapy.

Beside the perceived empathy of the therapist and the therapy motivation of the patient the education level and the depression level ad admission also showed to have a significant impact on the therapy outcome.

In other studies the influence of the education level on therapy outcome was contrarily discussed. On the one hand, in the study of Riedel (14) patients with a low education level showed a significantly lower reduction of symptoms in psychotherapy. However, on the other hand, Hiller et al. (15) showed that a lower education level correlated with better symptom improvement. Other studies could not detect any relationship between education and therapy success (6, 16). In our study the influence of the education level was significant, but not prominent compared to therapy motivation and therapist's empathy. Our study showed a significant relationship between the level of depressive symptoms ad admission and therapy outcome. This is in accordance with other studies also showing a higher benefit for depressed patients (2, 10, 21, 22).

## Limitations

In this study, a heterogeneous sample regarding main diagnoses was enquired, not especially depressive disorders. Nevertheless, we chose the improvement of depressive symptomatology as indicator of therapy success because almost all patients showed depressive symptoms ad admission.

One limitation constitutes that the perceived empathy was first assessed at the follow-up of the survey. Nevertheless, there was the risk of patients giving socially desirable answers to questions during the inpatient psychotherapy. Additionally there were multiple therapeutic relationships that exist during the inpatient psychotherapy which could be characterized by empathy. Patients often report in the Psy-BaDo that the relationship to a single psychotherapist is of utmost importance. This is congruent to the results of two other studies (23, 24), in which just the single session psychotherapy was a relevant factor for the success of inpatient psychotherapy.

Furthermore, there is a limitation because of the relative broad range of the length of the follow-up. However, in our regression analysis we could demonstrate that there is no significant impact of the length of the follow-up on the improvement of depressiveness. Additionally, Fliege et al. (12) showed that the time interval between discharge and follow-up does not predict the results of therapy at follow-up.

A further limitation reveals the heterogeneity of the patient sample which constitutes a strength of the study in terms of the naturalistic study design. Because of missing data it could not be investigated how the perceived empathy was assessed by patients who did not participate in the study or who did interrupt their therapy in an early stage. It remains to be seen whether perceived empathy has an impact on the termination of the treatment and on the refusal of study participation. Furthermore, it has to be critically remarked that patients with good therapy success estimate the therapeutic relationship better when compared with patients who have less therapy success. From our point of view this could be transferred on perceived empathy. On the one hand the initially perceived empathy could foster the development of a good therapeutic relationship and contribute to the therapy success. On the other hand after a successful therapy the assessment of perceived empathy could be influenced positively in retrospect. It has to be emphasized that perceived empathy was not assessed during the inpatient psychotherapy but rather at follow-up. Our results could speak for an internalization of object relationship to the therapist as a sustained therapy effect. Furthermore, we have to consider that therapy motivation in our study was only assessed by the therapist and was not supplemented by a self-rating questionnaire of the patient. The question has to be posed which aspects of the patients influenced the perception of the therapist concerning the assessment of motivation. Nevertheless, the results showed unequivocally that the therapy motivation of the patient reveals a clear success criterion.

## Conclusion

Therapy motivation of the patients at the beginning of the treatment as well as perceived empathy of the therapist by the patient seems to have a relevant impact on success of inpatient psychotherapy regarding the improvement of depressive symptomatology. Therapy motivation should therefore be assessed at the beginning of the therapy and be fostered by therapeutic procedures if necessary (17, 19).

Because of the strong influence of therapist's empathy the claim of other authors (27, 30) is justified to focus more on this ability in the postgraduate training of psychotherapists and in the context of supervision and special trainings. Summers presented a guide for a psychiatry residency psychodynamic therapy course (64). He described a model for teaching psychotherapy with empathy as important attitude for the building of a therapeutic alliance. It should be an essential aim for all psychotherapists to develop an empathetic attitude to the patient which could influence the therapy success in a positive manner.

## Data Availability

Datasets are available on request. The raw data supporting the conclusions of this manuscript will be made available by the authors, without undue reservation, to any qualified researcher.

## Author Contributions

FV, ST, and OO contributed to the conception and design of the study. FV and ST organized the database. MH, FV, ST, and OO performed the statistical analysis. FV and ST wrote the first draft of the manuscript. OO, HP, CA, and MH wrote sections of the manuscript. All authors contributed to the manuscript revision, read and approved the submitted version.

### Conflict of Interest Statement

The authors declare that the research was conducted in the absence of any commercial or financial relationships that could be construed as a potential conflict of interest.

## Footnotes

^1^DIMDI - OPS Operationen- und Prozedurenschlüssel http://www.dimdi.de/static/de/klassi/ops/index.htm (accessed Jul 29, 2018)
